# Ferritin-Based Nanocarriers: Analytical Methods for Physicochemical Characterization

**DOI:** 10.3390/ijms27209383

**Published:** 2026-10-21

**Authors:** Elena N. Subcheva, Alexandra A. Martynova, Darya S. Volkova, Andrey V. Zvyagin, Alessandro Parodi, Alexey V. Ermakov

**Affiliations:** Scientific Center for Translational Medicine, Sirius University of Science and Technology, 354340 Sirius, Russia; subcheva.en@talantiuspeh.ru (E.N.S.); zvyagin.av@talantiuspeh.ru (A.V.Z.); parodi.a@talantiuspeh.ru (A.P.)

**Keywords:** ferritin, biomimetic nanoplatform, nanoparticles, analytical methods

## Abstract

Unique physicochemical properties of ferritin nanoparticles, such as high biocompatibility, precise structural assembly, and modular surface chemistry make them a promising biomimetic platform for the delivery of therapeutic and diagnostic agents, including radioactive isotopes. However, the translation of ferritin from laboratory-scale synthesis to clinical applications remains hindered by the lack of standardized analytical workflows and requires precise qualitative and quantitative analysis of core loading, structural integrity, and long-term stability. This review systematically examines current characterization techniques ranging from morphological evaluation and quantitative elemental analysis to advanced spectroscopic and functional performance assays for evaluating metal and non-metal encapsulation efficiency, release kinetics, stability in complex biological media, and functional performance of ferritin-based nanocarriers. We highlight the correlation between the analytical metrics and biomedical outcomes, addressing the critical challenges in standardization necessary for clinical translation. The discussed methodologies establish a methodological roadmap for the rational design and quality control of ferritin-based theranostic systems, facilitating their path toward clinical implementation.

## 1. Introduction

Ferritin is a key protein that ensures the maintenance of intracellular iron homeostasis. Its primary function is the deposition of iron within its internal cavity in the form of an inorganic mineral core, which can contain up to 4500 Fe^3+^ ions, as well as the timely release of iron when needed through the process of “ferritinophagy” involving lysosomes [1].

**Figure 1:**
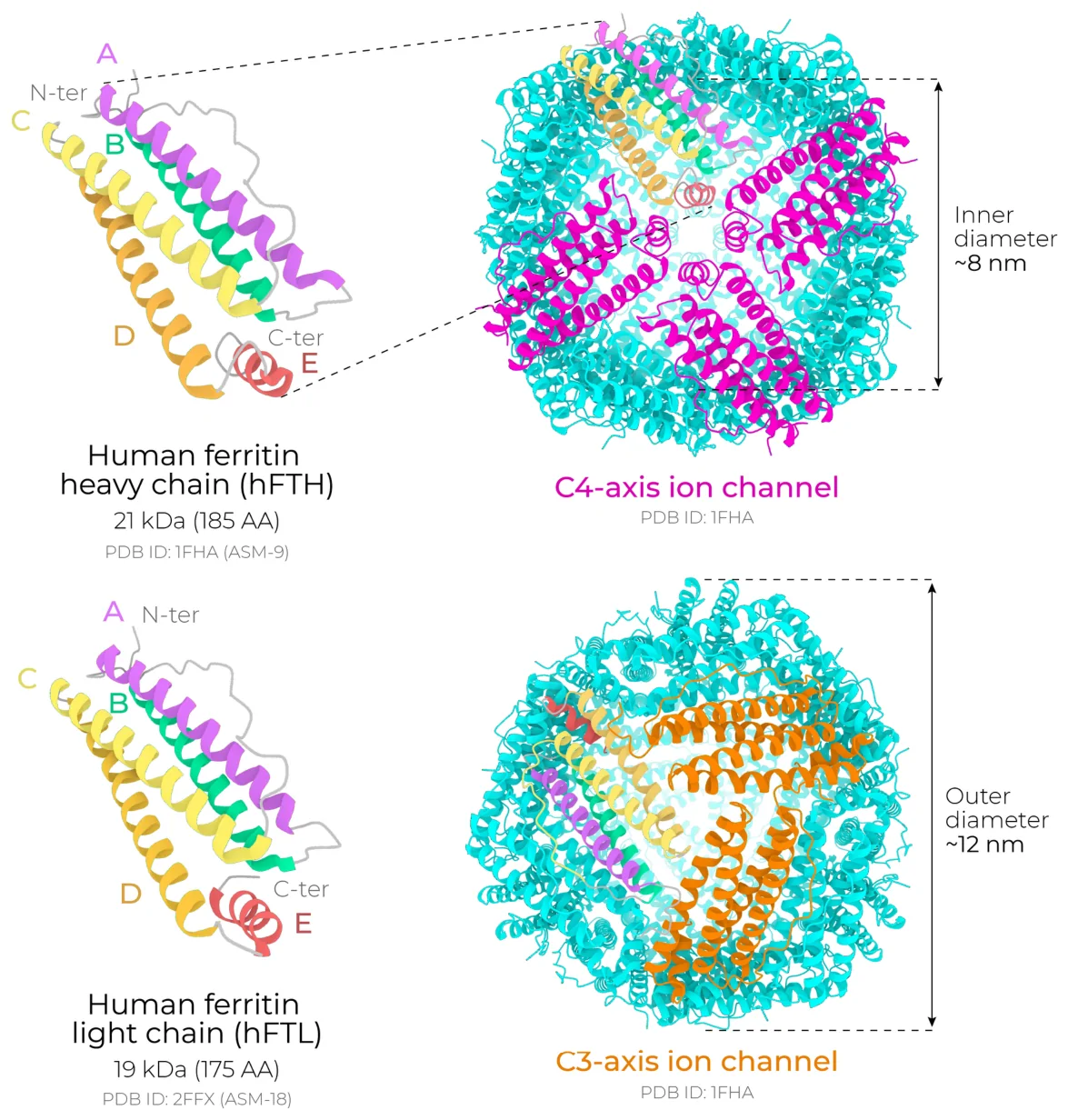
Structure of human ferritin. Left: schematic representation of the four-helix α-bundle of the heavy (H) and light (L) chains; helices A and D are approximately parallel to each other, helices B and C are antiparallel to them, and a short helix E is positioned at a 60° angle relative to the main bundle. Right: assembled 24-subunit octahedral cage viewed along the four-fold (C4) and three-fold (C3) axes, with the inner (~8 nm) and outer (~12 nm) diameters indicated. PDB IDs: 1FHA and 2FFX.

The structural framework of human ferritin is formed by two types of subunits—light (L) and heavy (H) chains. The light chain consists of 175 amino acids and has a molecular weight of approximately 19 kDa; it is encoded by the *FTL* gene (ferritin light chain, hFTL) located on the long arm of chromosome 19. The heavy chain comprises 185 amino acids, has a molecular weight of approximately 21 kDa, and is encoded by the *FTH1* gene (ferritin heavy chain 1, hFTH1) located on the long arm of chromosome 11. The three-dimensional organization of both chains is represented by a four-helix alpha-bundle (Figure 1, A–D): helices A and D are oriented approximately parallel, while helices B and C are antiparallel to them. A short helix E is positioned at a 60° angle relative to the main bundle, with each helix separated from the others by disordered regions of secondary structure [2].

The complete globular complex of human ferritin is formed by 24 subunits that assemble into a symmetric octahedral cage. Its outer diameter is 12 nm, and its inner diameter is 8 nm [2,3,4].

In recent years, ferritin nanoparticles have emerged as a promising biomimetic platform for biomedical applications owing to their unique properties, including the ability to encapsulate a broad spectrum of agents—ranging from metals and radionuclides to therapeutic drugs and nucleic acids [5,6,7,8,9,10]. Significant progress has been made in loading ferritin with bioactive compounds for applications in targeted radiotherapy [9,10], magnetic resonance imaging (MRI) contrast enhancement [11,12,13], and targeted drug delivery [3,5,6,7,8,14,15]. However, the rapid expansion of experimental studies in this field has given rise to a “methodological gap” that increasingly hinders data comparability and translational progress.

Despite the apparent simplicity of ferritin, the formation and long-term stability of encapsulated cargoes pose a considerable physicochemical challenge Several widely employed analytical techniques—including transmission electron microscopy [16,17], X-ray diffraction (XRD) [18,19], and inductively coupled plasma optical emission spectrometry (ICP-OES) [20,21]—are based on well-established physical principles and have been extensively validated in materials science. However, their reproducibility in the specific context of ferritin-based nanocarriers depends critically on sample preparation, instrumental settings, calibration, and data processing, and substantial inter-laboratory discrepancies persist at these stages. Importantly, fully standardized analytical workflows validated specifically for ferritin nanocarriers do not yet exist, and the absence of ferritin-specific reference materials and universally accepted acceptance criteria remains a significant obstacle to reproducibility and clinical translation [22,23,24]. The literature reflects a broad spectrum of methodological rigor, ranging from relatively simple approaches such as dynamic light scattering and ultraviolet–visible (UV–Vis) spectroscopy [22,23,24] to resource-intensive structural analyses, including cryo-electron microscopy and extended X-ray absorption fine structure (EXAFS) spectroscopy [19,25,26]. This heterogeneity hinders cross-study comparability and complicates the establishment of standardized characterization protocols. Moreover, key physicochemical parameters—including core composition, crystallinity, and loading efficiency—are only rarely correlated with downstream biomedical outcomes such as imaging contrast, therapeutic efficacy, or in vivo stability. This challenge is further compounded by the emergence of multi-agent systems that incorporate multiple metals [27] or combine organic and inorganic components [28], the characterization of which necessitates multimodal analytical strategies. Additional constraints arise from the resource demands of high-resolution techniques and the limited sensitivity of simpler methods when applied to low-concentration or structurally heterogeneous samples [29,30].

The present review seeks to address these gaps by providing a systematic examination of contemporary methods for the characterization of ferritin-based nanocarriers. It consolidates current knowledge on how diverse analytical approaches enable the evaluation of encapsulation efficiency for both metallic and non-metallic cargoes, release kinetics under physiologically relevant conditions, structural integrity of both the protein shell and the encapsulated core, and functional performance—including MRI relaxivity and radiotherapeutic cytotoxicity. By correlating analytical metrics with biomedical outcomes, this review aims to identify best practices and delineate the key standardization challenges that must be overcome to advance ferritin-based platforms toward clinical application.

## 2. Methods for Characterization of Ferritin-Based Nanocarriers

Accurate characterization of the inner core of ferritin nanoparticles is a critical step in the development of biomedical nanocarriers. The functional performance of these nanoparticles, including the efficiency of bioactive agent delivery, release kinetics, MRI contrast properties, and radiotherapeutic activity in the case of radionuclides, directly depends on the reliable assessment of core composition, structure, crystallinity, and stability [31,32].

Modern analytical techniques enable comprehensive characterization of the ferritin core. The choice of a specific method is dictated not only by the nature of the encapsulated cargo, whether inorganic compounds such as Ga_2_O_3_, Lu_2_O_3_, Gd_2_O_3_, Fe_3_O_4_, MnO_2_, and other oxides or metals, or organic molecules including drugs, nucleic acids, and peptides [11,14,15,21,33,34], but also by the specific analytical question being addressed, the expected analyte concentration, the required sensitivity, the sample matrix, and whether qualitative or quantitative information is needed. For example, the determination of metal loading in a single ferritin particle requires a technique with high elemental sensitivity and a low detection limit, such as inductively coupled plasma mass spectrometry (ICP-MS), whereas the identification of a crystalline phase within the core may be adequately addressed by XRD or electron diffraction, depending on the crystallite size and degree of amorphization. Similarly, the analysis of organic cargoes in complex biological media demands methods capable of discriminating between encapsulated and free molecules, which places different requirements on selectivity and matrix tolerance than the analysis of purified samples in simple buffers. A systematic consideration of these factors is therefore essential for selecting an appropriate analytical strategy and for ensuring that the resulting data are interpretable and comparable across studies.

For inorganic cores, the primary analytical objectives include determining the phase composition and degree of crystallinity, assessing the size and morphology of nanoparticles confined within the ferritin cavity, quantifying metal loading, probing the local coordination environment and oxidation state of metal atoms, and monitoring core stability under physiologically relevant conditions [18,35]. For organic cores, different priorities prevail: confirming encapsulation without compromising the integrity of the protein shell, evaluating loading efficiency and the uniformity of cargo distribution within the cavity, characterizing potential interactions between the encapsulated molecules and the inner surface of the nanoparticle, and investigating release kinetics in model biological media [36,37].

This section presents the most informative methods for ferritin core analysis, grouped according to their underlying physical principles. For each method, the specific analytical tasks relevant to ferritin-based nanoparticles are described, along with inherent limitations and potential artifacts encountered when analyzing biomedical samples, and representative examples of successful application from recent studies are provided. Particular emphasis is placed on the importance of a multimodal analytical approach: the combination of complementary techniques—such as transmission electron microscopy (TEM) with X-ray diffraction (XRD), or ICP-MS with Raman spectroscopy—provides a more complete picture and helps avoid ambiguous data interpretation [38]. Such a multidisciplinary analytical framework is a prerequisite for the standardization of characterization protocols for ferritin-based nanocarriers prior to preclinical and clinical evaluation. This is particularly relevant for the development of radionuclide delivery systems, MRI contrast agents and targeted therapeutic nanoparticles, where the accuracy of core characterization directly influences the safety and efficacy of the final biomedical product.

### 2.1. Transmission Electron Microscopy

TEM enables imaging with a focused electron beam at near-atomic resolution. Image contrast is governed by the atomic number, density, and thickness of the sample, as well as by the accelerating voltage [39]. For organic specimens, lower voltages improve contrast in ultrathin samples, whereas higher voltages with thicker films enhance resolution. At constant voltage, increasing the atomic number (Z) per unit volume enhances the effective scattering cross-section, while the concentration of heavier atoms influences scattering intensity [40]. When sample atoms have comparable scattering power, contrast arises from differences in the mass-thickness parameter between adjacent regions [40]. Since N, C, and O exhibit nearly identical scattering abilities, contrast must originate from variations in thickness or density within the specimen [39].

TEM is a widely used analytical technique that enables the investigation of ferritin particle morphology, particle and core dimensions, the presence of impurities, and nanoparticle agglomeration. Ferritin was first visualized by J. L. Farrant in 1954 [41]. To obtain micrographs, ferritin was purified by fivefold recrystallization from a 6% cadmium sulfate solution, after which the crystals were dissolved in distilled water and dialyzed to remove electron-dense cadmium ions. The resulting suspension was deposited onto support grids and examined under two conditions: without staining and with contrasting using uranium salts. This pioneering study provided the first characterization of iron-containing cores, some of which exhibited a distinctive four-sector internal structure, and established the iron core diameter range (27–55 Å) as well as the internal cavity diameter of approximately 95 Å. These findings definitively ruled out hypotheses suggesting that ferritin accumulates iron in regions other than the central cavity of the particle.

**Figure 2:**
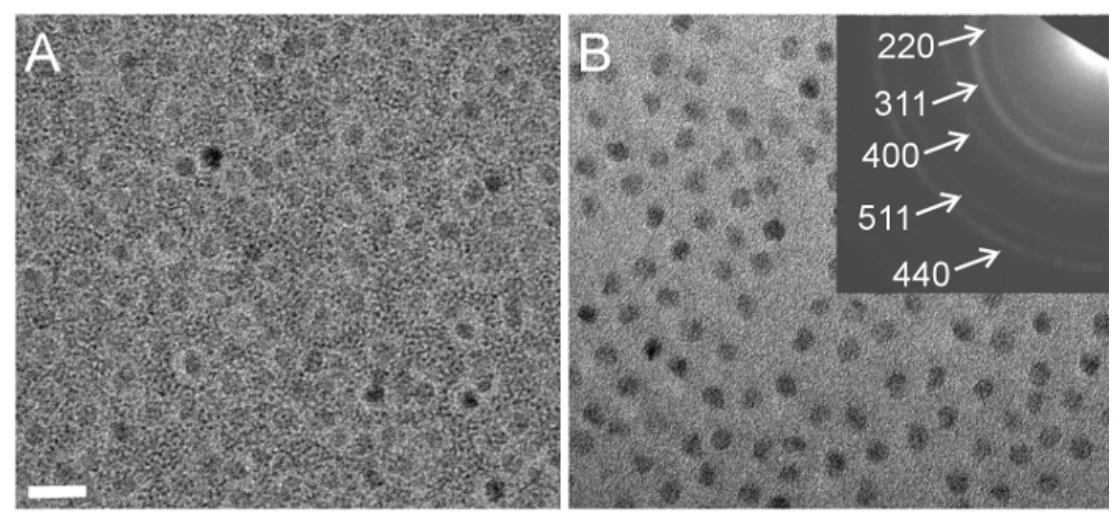
TEM images of magnetoferritin stained with aurothioglucose (**A**) and unstained (**B**). The inset shows the corresponding electron diffraction pattern with magnetite indices; the arrows indicate the (220), (311), (400), (511), and (440) reflections. The scale bar of 20 nm is shown in panel A and applies to both panels. Adapted from ref. [42].

For the characterization of ferritin nanoparticles loaded with metals or metal oxides, negative staining with a 1% aurothioglucose solution can be employed. Due to its large molecular size, aurothioglucose cannot penetrate the interior of the ferritin cage, thus providing strong surface contrast. This enables the assessment of the degree of cargo loading—analogous to the enumeration of empty versus full viral particles—as well as the determination of core size and crystallinity (or amorphous nature). An example of such negative staining is presented in Figure 2 [42].

Modern scanning transmission electron microscopy (STEM) imaging, particularly in high-angle annular dark-field (HAADF) mode, provides high contrast and resolution, enabling detailed studies of iron-containing core formation [43,44]. This capability largely arises from the sensitivity of the method to two parameters: the atomic number of the element (Z-contrast) and the sample thickness [45].

**Figure 3:**
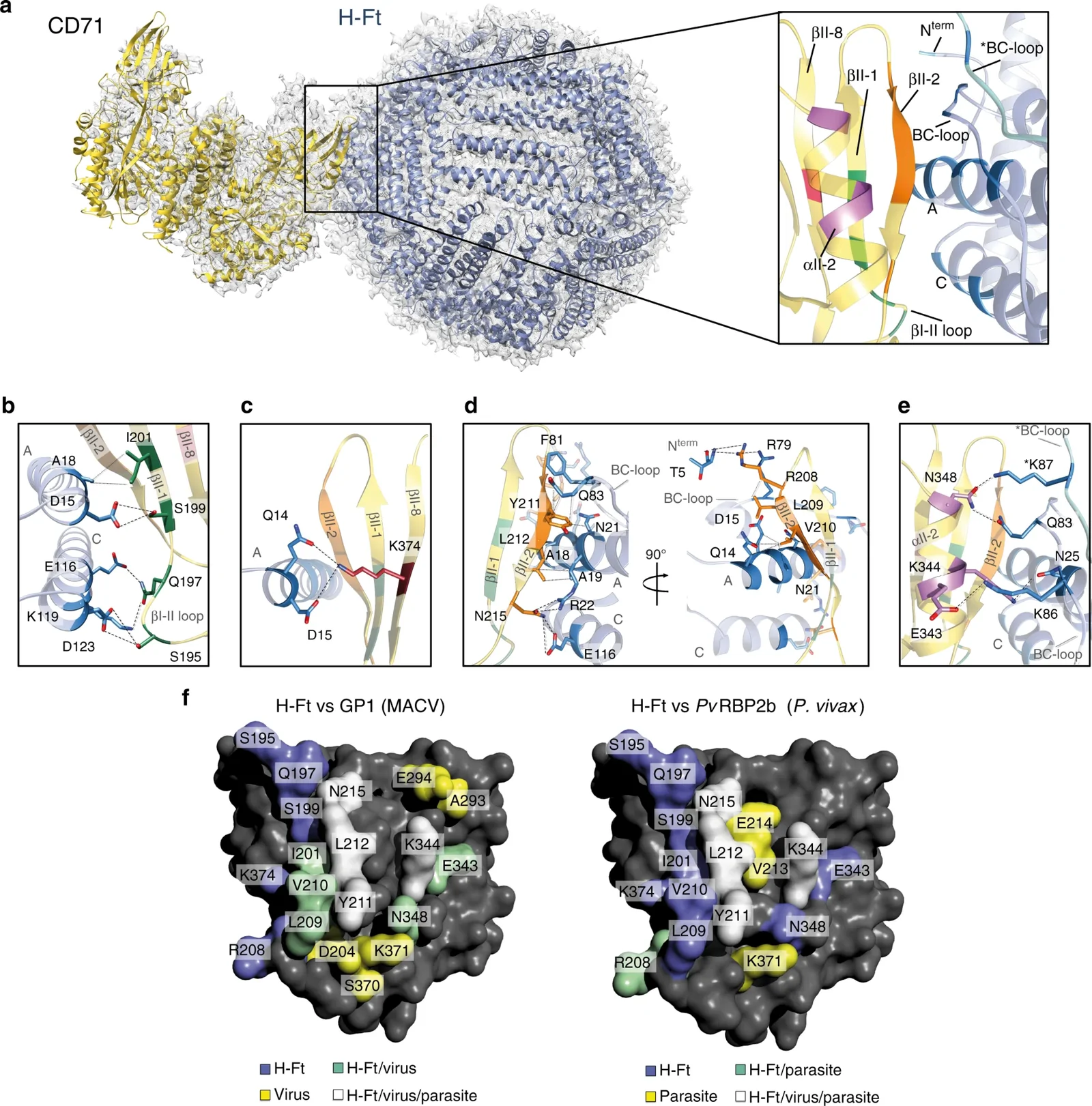
Structure of the human CD71/H-Ft complex. (**a**) Atomic model of the complex of human CD71 receptor (yellow) and human H-Ft (light blue) fitted in the cryo-EM map at a global resolution of 3.9 Å (gray mesh) is shown. On the side, close-up view of the contact region. The interacting residues of CD71 are highlighted by different colors depending on the specific secondary structural element of the apical domain to which they belong; the interacting residues of H-Ft are colored in light blue. (**b**) The βII-1 strand and the βI-1–βII-1 loop of CD71 (S195, Q197, S199, I201) interact with the A helix (Q14, D15) and the C helix (E116, K119, D123) of H-Ft. (**c**) The βII-8 strand of CD71 (K374) forms an electrostatic interaction with Q14 at the N-terminus of the H-Ft A helix. (**d**) “Common contacts” between CD71 and H-Ft: the βII-2 strand of CD71 (Y211, L212, N215) interacts with the H-Ft A helix, the BC-loop (the loop connecting the B and C helices; F81, Q83) and the N-terminus (T5, N21, A18, A19, R22), while the αII-2 helix of CD71 interacts with the A helix and the BC-loop (K87) of a flanking H-Ft monomer; the C helix (D15, E116) and the N-terminus (Q14, N21) are also involved. (**e**) Additional “common contacts”: the αII-2 helix of CD71 (E343, K344, N348) interacts with the BC-loop (Q83, K86) and the N-terminus (N25) of H-Ft. Contacting residues within 5 Å are shown as sticks and labeled; dashed black lines indicate electrostatic interactions, and dotted gray lines represent hydrophobic contacts. (**f**) Surface representation of the human CD71 apical domain (dark gray). Left: residues contacted by human H-Ft and by GP1 of Machupo virus (MACV) (S195, Q197, S199, I201, L209, V210, Y211, L212, N215, R208, D204, S370, K371, K374, E343, K344, N348, A293, E294). Right: residues contacted by human H-Ft and by PvRBP2b of *P. vivax* (S195, Q197, S199, I201, L209, V210, Y211, L212, N215, V213, R208, K344, E343, K371, K374, N348), mapped according to the color code shown at the bottom. Adapted from ref. [47].

The advent of cryo-electron microscopy (cryo-EM) has further enabled structural investigations of ferritin particles and their interactions with various molecules [46]. For instance, a model of the ferritin–transferrin receptor 1 (CD71) complex was obtained by fitting cryo-EM projection images (Figure 3) [47]; the structure of ferritin bearing a light-chain mutation was investigated [48]; and the boundary between the protein shell and the encapsulated iron oxide phases was directly visualized [49,50].

The crystallinity of ferritin cores can be assessed using electron diffraction modes, including nanodiffraction, as demonstrated by Quintana et al. [51]. Electron diffraction enables the identification of crystalline phases within the cores. For example, Chaudhary et al. [52] employed selected-area electron diffraction (SAED) to evaluate the crystallinity of CdSe:CdS:ZnS quantum dots and confirmed their encapsulation within apoferritin via high-resolution TEM (HRTEM) with negative staining.

In summary, the most common applications of transmission electron microscopy include the examination of core crystallinity (particularly for metals or metal oxides), confirmation of the presence of ferritin nanoparticles, detection of impurities and aggregation, and assessment of particle loading or verification of encapsulation. Negative-stain TEM cannot be regarded as an independent method for determining loading efficiency and, in this context, serves exclusively as a complementary technique to the primary quantitative method, such as ICP-MS or ICP-OES for metal-loaded ferritin, or high-performance liquid chromatography (HPLC) and UV–Vis spectrophotometry for organic cargoes. In addition, negative-stain TEM can provide supporting evidence for the preservation of the ferritin structure and for the localization of the cargo within the internal cavity rather than on the outer surface of the protein shell, albeit with certain caveats related to staining, drying, particle orientation, and image-selection bias.

### 2.2. X-Ray Analysis

#### 2.2.1. X-Ray Diffraction (XRD)

XRD is a technique for investigating the structure of materials based on the diffraction of X-rays, enabling qualitative and quantitative phase analysis, structure determination and refinement, and assessment of micro- and macrostrain. The two most widely used variants are single-crystal X-ray diffraction (SC-XRD), employed for determining lattice parameters, symmetry, and atomic positions within the unit cell, and powder X-ray diffraction (PXRD), used for the identification and quantification of polycrystalline phases [53,54].

Early X-ray powder diffraction studies of ferritin by Fankuchen [55] demonstrated that ferritin and apoferritin adopt cubic lattices and are isomorphous. Subsequent crystallographic investigations by Hodgkin and Cowan revealed an orthorhombic structure and an iron core diameter of approximately 5 nm [56]. In 1959, Harrison [56] determined the molecular diameter to be 111–112 Å and established that the unit cell, containing four molecules, exhibits cubic 432 point symmetry and is assembled from 24 identical subunits, consistent with a near-spherical architecture and earlier electron microscopy observations. In later work, Harrison [57] proposed that ferritin crystals contain molecules with point symmetry lower than 432 and suggested that apoferritin might adopt 532 or pseudo-532 (pseudo-icosahedral) symmetry, i.e., consist of 20 subunits arranged at the vertices of a pentagonal dodecahedron. However, this structural model was refuted by Hanson in 1968 [58], who demonstrated that the subunits could not occupy all equivalent positions within such an arrangement, as this would require either two distinct subunit types or the same subunit in two different conformations, neither of which was supported by experimental data. This led to the re-establishment of the 24-subunit model of apoferritin [59]. In 1975, Hoare et al. [60] investigated metal-ion binding sites in ferritin and generated a 6 Å resolution electron density map using Cd^2+^ and UO_2_^2+^ as markers, resulting in a spherical structural model with a cubic lattice. Subsequent studies based on these data [61] determined the internal and external diameters (7–9 nm and 12–13 nm, respectively), refined the model from a perfect sphere to a near-spherical shape, and estimated the minimal channel dimensions based on the sizes of molecules penetrating the apoferritin shell.

Results obtained by Heald et al. [62] using extended X-ray absorption fine structure (EXAFS) spectroscopy in 1979 provided the first detailed structural insights into the iron-containing core of ferritin. The study demonstrated that only a two-dimensional layered model with hexagonal symmetry was consistent with the experimental data. In this model, the layers consist of planes of Fe atoms separated by close-packed O (or OH) species, with phosphorus acting as a “bridging” agent that defines the spacing between the layers. This structural organization accounts for both the stability of the iron storage core and its capacity for controlled iron release.

EXAFS spectra are typically acquired using synchrotron radiation, and the method is based on measuring the energy-dependent X-ray absorption coefficient of a material [63]. This technique enables structural investigations of biological molecules containing metal ions, providing information on the electronic structure of the metals and their local coordination environment.

Currently, X-ray powder diffraction is widely employed to identify and confirm the phase composition of metallic or oxide nanoparticles synthesized within ferritin templates [19,64]. In most studies, XRD analysis is performed after thermal treatment of ferritin (typically at approximately 400 °C), which removes the protein shell and leaves only the inorganic core nanoparticles. This approach enables the unambiguous identification of oxide phases or, in the case of platinum-group systems, metallic phases in their pure form. However, it should be emphasized that such thermal treatment may not only remove the protein shell but also alter the original inorganic phase, crystallinity, oxidation state, or particle size of the core. Consequently, the resulting diffraction pattern may not necessarily represent the native ferritin core, and the phase composition determined after thermal treatment should be interpreted with caution and, where possible, validated by complementary techniques that do not require removal of the protein shell, such as electron diffraction, X-ray absorption spectroscopy, or Raman spectroscopy.

#### 2.2.2. Small-Angle X-Ray Scattering (SAXS)

SAXS is one of the most widely used techniques for investigating soft materials, including various biomaterials and polymers, as well as macromolecules in solution, by measuring the scattering contrast between the macromolecules and the surrounding buffer with high resolution [65]. SAXS provides information on molecular size and shape, which can be used to generate low-resolution structural models, and enables studies of self-assembly processes. For example, the kinetics of ferritin self-assembly have been investigated by laser light scattering as a function of subunit concentration, pH, and ionic strength, and time-resolved SAXS has been applied to elucidate the mechanisms of ferritin assembly [66]. Additionally, low-resolution structural studies of apoferritin using both small-angle neutron scattering (SANS) and SAXS have examined the effect of protein concentration [67].

McMath et al. [68] combined SAXS data obtained for *Mycobacterium tuberculosis* BfrB in solution with subsequent superposition of the averaged electron density envelope (derived from 16 independent calculations using CHIMERA) onto the crystal structure of a homologous ferritin (*T. maritima*, PDB 1vlg). This analysis led to the conclusion that Mtb BfrB assembles into a 24-subunit spherical complex. The hollow nature of the resulting envelope further indicated the absence of iron in the Mtb BfrB sample. Thus, the combination of SAXS and computational modeling not only confirmed the expected architecture of the protein complex but also provided insight into its functional state.

**Figure 4:**
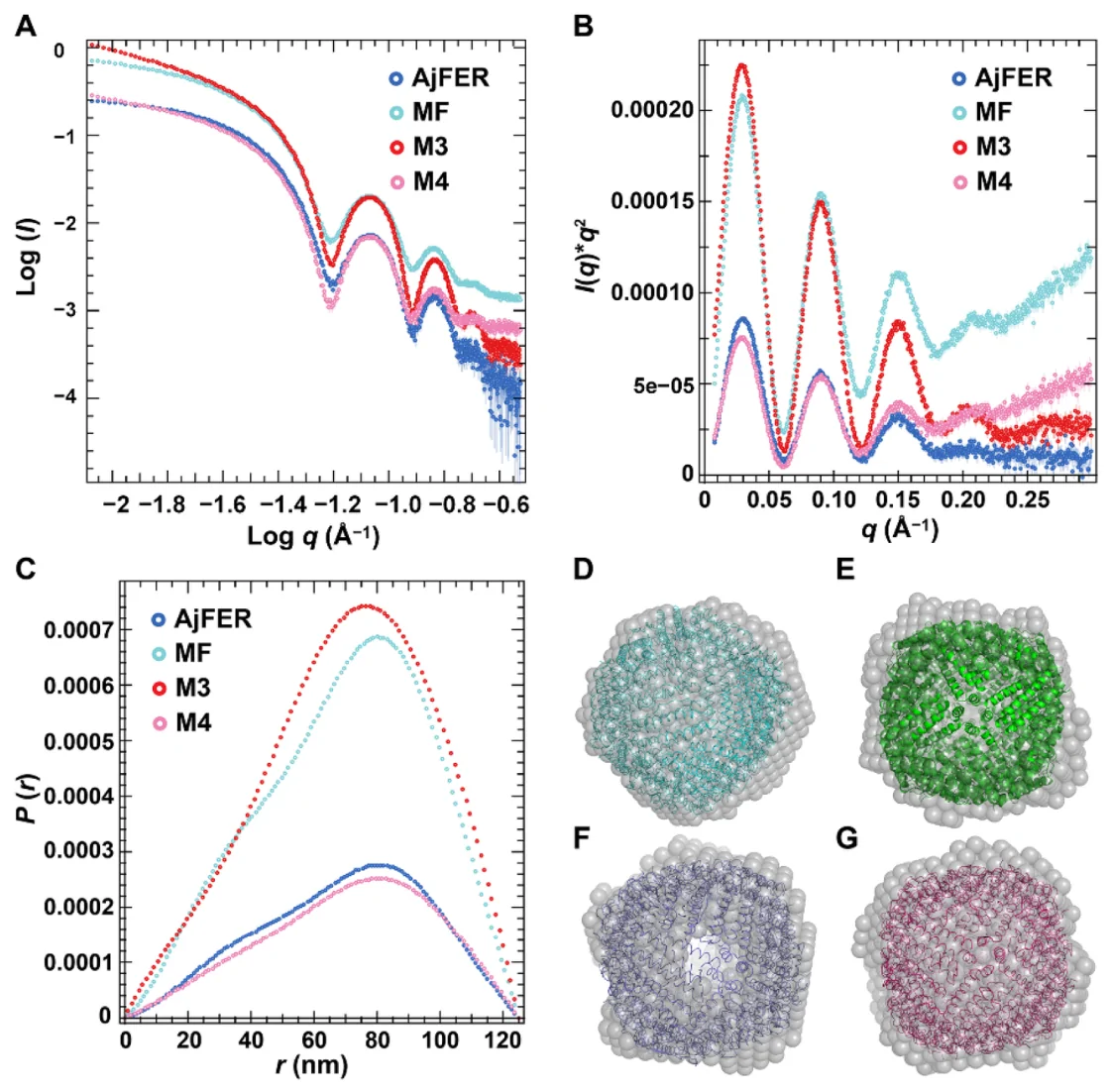
SAXS analysis of AjFER and its variants. (**A**) Experimental SAXS scattering curves of AjFER (blue circles), MF (light blue circles), M3 (red circles), and M4 (pink circles); the curves are plotted as log *I* versus log *q*(Å^−1^). (**B**) Kratky plot analysis of the same samples, shown as *Iq*^2^ versus *q*(Å^−1^). (**C**) Pair distance distribution function P(*r*) versus distance *r* (nm) for AjFER, MF, M3, and M4. (**D**) Superposition of the averaged *ab initio* SAXS envelope (gray) of AjFER with the crystal structure of AjFER (PDB ID: 7VHR). (**E**) Superposition of the averaged *ab initio* SAXS envelope (gray) of the E25A/E60A/E105A mutant (MF) with the crystal structure of AjFER (PDB ID: 7VHR). (**F**) Superposition of the averaged *ab initio* SAXS envelope (gray) of the D129A/E132A mutant (M3) with the crystal structure of AjFER (PDB ID: 7VHR). (**G**) Superposition of the averaged *ab initio* SAXS envelope (gray) of the E168A mutant (M4) with the crystal structure of AjFER (PDB ID: 7VHR). Adapted from ref. [69].

A representative extension of this approach is provided by Wu et al. [69], who characterized *Apostichopus japonicus* ferritin and its mutants with substitutions at the ferroxidase center, the three-fold channel, and the four-fold channel. SAXS data processed with PRIMUS, GNOM, and DAMMIF/DAMMIN revealed profiles typical of hollow spherical particles, with Rg (52.6–53.8 Å) and Dmax (120–126 Å) matching the expected dimensions of a 24-meric cage (Figure 4). Ten independent ab initio bead models superimposed with the crystal structure of AjFER (PDB 7VHR) fitted well into the SAXS envelopes, and the theoretical scattering curve agreed with experimental data (χ^2^ = 52.7 by FoXS). Notably, for mutant proteins, discrepancies between crystal and solution structures were detected, indicating that SAXS captured subtle conformational changes not visible in the crystal lattice.

SAXS can also be applied to investigate dynamic processes and structural changes induced by variations in external conditions. For example, Sato et al. [66] studied the pH-dependent assembly of *Escherichia coli* ferritin using time-resolved SAXS (TR-SAXS). It was established that under acidic conditions, the ferritin cage dissociates predominantly into dimers, which retain their native secondary and tertiary structures and are capable of reassembling into the 24-meric complex upon a rapid increase in pH. This reversible behavior can be exploited for loading cargoes into ferritin that cannot be encapsulated by other methods. Modeling of the assembly process also revealed the formation of tetramers, hexamers, and dodecamers as intermediates [66,70]. Thus, TR-SAXS enabled not only the detection of intermediate states but also the quantitative characterization of their contributions to the overall assembly mechanism.

SAXS also enables the detection of “dead-end” oligomeric states, as demonstrated in a study by Sudarev et al. [71]. In that work, the ferritin subunit was modified with the SMT3 protein tag, a homolog of the human small ubiquitin-like modifier (SUMO-tag), and the self-assembly process was investigated by combining SAXS data with AlphaFold-based modeling. This approach revealed the formation of stable octameric oligomers that did not progress to fully assembled 24-meric globules.

The investigation of ferritin cores by SAXS remains challenging; therefore, this method is primarily applicable to studies of the dynamic behavior of ferritin or the characterization of its individual oligomeric forms rather than direct core analysis.

### 2.3. Spectroscopic Methods

#### 2.3.1. Raman and FTIR Spectroscopy

Raman spectroscopy is an analytical technique based on the inelastic scattering of monochromatic light. When a sample is irradiated with a laser, a small fraction of the incident photons interacts with molecular vibrations and undergoes a change in energy, a phenomenon known as the Raman effect [72]. In the context of loaded ferritin, Raman spectroscopy can be employed to distinguish between different mineral phases, monitor conformational changes in the protein shell, assess the degradation of organic cargo, and detect structural alterations of encapsulated organic molecules after loading [73]. Complementary information on functional groups and molecular interactions can be obtained by Fourier-transform infrared (FTIR) spectroscopy, which probes dipole-allowed vibrational transitions and is particularly useful for confirming the presence of specific chemical bonds in both the protein shell and the encapsulated cargo.

Hartmann et al. [73] demonstrated the capabilities of Raman microspectroscopy (RM) for the nondestructive analysis of the chemical composition and structure of the iron-containing core of ferritin. The authors synthesized reference nanoparticles of ferrihydrite (Fe_2_O_3_·0.5H_2_O), maghemite (γ-Fe_2_O_3_), and magnetite (Fe_3_O_4_), optimized RM parameters (laser wavelengths and power), and compared the Raman spectra of ferritin and apoferritin with those of the reference minerals; the results were further validated by Mössbauer spectroscopy and X-ray diffraction. A characteristic broad band at approximately 730 cm^−1^ was observed in the ferritin spectrum but was absent from that of apoferritin, indicating the presence of a ferrihydrite-like phase in the protein core. For magnetite, a strong narrow band at 665 cm^−1^ and a weak band at 540 cm^−1^ are typical, while maghemite exhibits a broad band near 700 cm^−1^ with two maxima (668 cm^−1^ and 715 cm^−1^) and bands of medium intensity at approximately 370 cm^−1^ and 500 cm^−1^. In addition, amide I (~1650 cm^−1^) and amide III (~1300 cm^−1^) bands, characteristic of α-helical protein structure, as well as bands of aromatic amino acid residues, were detected in the spectra of both ferritin and apoferritin, enabling assessment of the conformational state of the protein shell. Thus, RM offers several advantages for ferritin analysis: measurements can be performed at room temperature without complex sample preparation, with high spatial resolution, under in situ conditions, and allow simultaneous investigation of both the mineral core and the protein shell.

FTIR spectroscopy was employed by Mao et al. [74] to assess the structural features of ferritin nanoparticles modified with ascorbic acid (ferritin–ascorbic acid nanoparticles, FANs). In particular, FTIR analysis revealed an increased number of iron-binding sites compared to native ferritin. Changes in the secondary structure of the protein within FANs, specifically increased disorder, were evaluated by circular dichroism spectroscopy. These structural modifications contribute to the efficient and controlled release of iron, thereby enhancing its bioavailability.

#### 2.3.2. X-Ray Photoelectron Spectroscopy (XPS)

XPS is a surface-sensitive analytical technique based on the photoelectric effect. When a sample surface is irradiated with monochromatic X-rays, electrons from the inner shells of atoms absorb photon energy and are ejected from the sample [75]. The spectrometer measures the kinetic energy of these photoelectrons, from which the electron binding energy is calculated. Since the binding energy is characteristic of each element and depends on its chemical environment, including oxidation state and bonding with neighboring atoms, XPS enables not only the identification of elements present on the surface but also the determination of their chemical states [76].

Using this method, Zhang et al. [77] confirmed the chemical reduction of Pt(II) to Pt(0) within the cavity of apoferritin. The Pt 4f_7/2_ and Pt 4f_5/2_ peaks shifted from 77.9 and 81.3 eV to 73.1 and 76.5 eV, respectively, corresponding to a shift of approximately 5 eV. Notably, the Pt 4f binding energy in the Pt–apoferritin system was found to be almost 2 eV higher than that of bulk platinum (71.2 eV for Pt 4f_7_/_2_), but 1.5 eV lower than that of PtO or PtO_2_ (74.6 eV). This observation suggests either an extremely small size of the platinum nanoparticles or potential interactions between the nanoparticles and the apoferritin protein shell. In either case, the data confirm the successful formation of active platinum nanoparticles within the protein structure without complete passivation of their surfaces.

When applied to ferritin, XPS is not a method of choice for the analysis of intact nanoparticles, since its surface sensitivity (5–10 nm) limits the detection of species located inside the intact protein cage. For this reason, XPS is primarily used for the characterization of inorganic compounds, particularly metals and metal oxides, after removal of the protein shell by plasma treatment, UV-ozone oxidation, or thermal treatment [77,78]. In this context, the main limitations are surface sensitivity, vacuum requirements, possible radiation damage to organic constituents, and the inability to provide information about the material located inside an intact protein cage [79].

#### 2.3.3. Energy-Dispersive X-Ray Spectroscopy (EDX)

Energy-dispersive X-ray spectroscopy (EDS or EDX) is an analytical technique that, in most cases, provides qualitative confirmation of the presence of elements based on their characteristic X-ray emission spectra generated by the interaction of an electron beam with the sample [80]. EDX is most commonly employed in combination with transmission electron microscopy (TEM-EDX) [15,81,82] or scanning electron microscopy (SEM-EDX) [83].

TEM-EDX was employed by Wang et al. [15] to confirm the chemical composition and structure of CuS–Fn nanocapsules. Specifically, the analysis demonstrated the successful formation of copper sulfide (CuS) within the ferritin (Fn) cavity, verified the localization of copper (Cu) and sulfur (S) in the core of the nanostructure, excluded the presence of contaminants, and enabled estimation of the Cu:S ratio to confirm CuS stoichiometry. Semi-quantitative analysis of the EDX spectrum revealed a Cu:S ratio close to 1:1, consistent with the expected stoichiometry of CuS. Comparison with a control Fn sample ruled out potential artifacts and confirmed the specificity of CuS loading within the ferritin cavity.

In a study by Affatigato et al. [83], SEM-EDX was applied to confirm the structure of the HumAfFt–SPIONs nanocomplex (humanized *Archaeoglobus fulgidus* ferritin–superparamagnetic iron oxide nanoparticles). The analysis revealed that the nanoparticle aggregates were coated with an organic material containing carbon and oxygen, corresponding to the ferritin protein shell, while iron was not detected on the particle surface. This finding confirmed the localization of SPIONs within the core of the nanostructure. Furthermore, comparative analysis with unmodified SPIONs allowed the determination of the iron-to-oxygen ratio (89.8% and 10.2%, respectively) and confirmed the successful application of the protein coating.

TEM-EDX remains the preferred method for the localization and qualitative confirmation of the elemental composition of loaded ferritin cores. Owing to the use of thin electron-transparent specimens and the high spatial localization of the analytical signal, TEM-EDX enables reliable detection of elements directly within the nanoparticle cavity, which is critical for objects approximately 12 nm in size. This method allows verification of the stoichiometry of encapsulated compounds and helps exclude artifacts through comparison with control samples. Quantitative or semi-quantitative EDX analysis is possible under appropriate calibration and acquisition conditions; however, at the level of individual ferritin nanoparticles, accuracy is strongly affected by low signal intensity, background, specimen thickness, geometry, and support-film contributions. Semi-quantitative estimates based on normalization of characteristic peak intensities are therefore more realistic in this context.

In contrast, SEM-EDX is characterized by a significantly greater X-ray generation depth (in the micrometer range, determined by beam energy and sample density), causing the signal to be averaged over a volume exceeding the size of an individual particle. Therefore, SEM-EDX is primarily suitable for assessing the composition and homogeneity of coatings and aggregates, as well as for confirming the localization of heavy elements within large agglomerates. However, the localization of elements within the core of an individual ferritin nanoparticle remains beyond the capabilities of SEM-EDX.

#### 2.3.4. Electron Energy-Loss Spectroscopy (EELS)

Electron energy-loss spectroscopy (EELS) is an analytical technique performed within a transmission electron microscope that measures the energy distribution of electrons after they have interacted with a thin specimen. When the incident electron beam passes through the sample, a fraction of the electrons undergoes inelastic scattering events, losing energy through excitations of plasmons, interband transitions, and inner-shell ionizations. By analyzing the energy-loss spectrum, EELS provides information on the elemental composition, electronic structure, oxidation state, and local coordination environment of the probed atoms with near-atomic spatial resolution. When combined with scanning transmission electron microscopy (STEM) in high-angle annular dark-field (HAADF) mode, EELS enables spatially resolved chemical mapping at the single-nanoparticle level, which is particularly valuable for analyzing the mineral core confined within the ferritin cavity [44,84].

For ferritin-based nanocarriers, EELS serves as a powerful complementary technique to EDX, providing not only qualitative and semi-quantitative elemental analysis but also chemical-state information that is inaccessible to X-ray-based methods. The technique is effective for a broad range of elements, including transition metals (Fe, Co, Cu, Mn, Gd, Lu), noble metals (Au, Pt, Pd), and lighter elements (C, N, O, S, Se), where the fine structure of the ionization edges—specifically the energy-loss near-edge structure (ELNES)—reflects the oxidation state and coordination geometry of the probed atoms. This versatility makes EELS applicable to ferritin loaded with diverse inorganic and organic cargoes, where the chemical state of the encapsulated agent directly influences the magnetic, catalytic, radiotherapeutic, or drug-release properties of the resulting nanocarrier.

The elemental composition and oxidation state of iron in ferritin cores have been extensively studied by EELS. Early applications focused on structural and compositional analysis of isolated horse-spleen ferritin particles without additional staining. Beckers et al. [85] employed energy-filtering transmission electron microscopy (EFTEM) combined with EELS to analyze ferritin particles collected in ultrathin chromium films, visualizing the proteinaceous shell through carbon net-intensity images and performing quantitative analysis of the number of carbon atoms in the shell. Because ferritin nanoparticles produce a weak EDX signal, Graham et al. [86] relied on EELS for identification of ferritin iron in organ tissues, using Fe L_2_,_3_ edge spectra to confirm the iron content of individual nanoparticles and demonstrating the spatial and temporal association of ferritin with invader nanoparticles in lung, liver, spleen, and brain.

Quantitative EELS analysis has enabled precise determination of iron oxide phases within ferritin. Walls et al. [87] used EELS to unequivocally identify magnetite (Fe_3_O_4_) as the iron oxide formed within recombinant human H-chain ferritin cores under specific preparation conditions. Atom counting analysis by EELS and HAADF imaging allowed correlation of particle sizes to actual Fe atom numbers in a quantitative manner, helping to clarify structural confusion between magnetite and maghemite (γ-Fe_2_O_3_). Longo et al. [44] combined HAADF-STEM, EELS, and ^57^Fe Mössbauer spectroscopy to investigate iron-reconstituted and genetically engineered human heteropolymer ferritins, revealing that H-rich proteins exhibit elongated, dumbbell, and crescent-shaped cores, while L-rich proteins present spherical cores, pointing to a correlation between core shape and protein shell composition.

The oxidation state of iron in ferritin cores has been a subject of particular interest. Using EELS, researchers have determined that iron in ferritin cores within human liver biopsies is entirely Fe^3+^ in octahedral coordination with oxygen, similar to the mineral ferrihydrite [88]. However, prolonged exposure to the electron beam can reduce Fe^3+^ to Fe^2+^ and induce phase transformations. The percentage of octahedrally coordinated Fe^3+^ decreases as the accumulated electron dose increases, and Fe^2+^ is produced in liver biopsies when the electron dose exceeds 10^6^ electrons/nm^2^ [88]. EELS has also been applied to study ferritin cores in diseased brain tissue: Quintana et al. [51] characterized ferritin molecules associated with tau filaments from patients with progressive supranuclear palsy and Alzheimer’s disease, finding significant differences in mineral composition between physiological and pathological ferritin cores.

Beyond iron-containing cores, EELS has been successfully applied to ferritin loaded with other inorganic materials. Tsukamoto et al. [89] synthesized cobalt oxide (Co_3_O_4_) nanoparticles within the cavity of apoferritin and used EELS to confirm the presence of cobalt atoms within the protein core. The EELS spectra, acquired alongside XPS data, provided direct evidence that the cores contained cobalt, complementing structural characterization by high-resolution TEM and XRD.

Despite its capabilities, EELS for ferritin analysis faces significant limitations. The most critical is electron-beam-induced radiation damage, which manifests as radiolysis, leading to reduction of metal ions (e.g., Fe^3+^ to Fe^2+^) and potentially producing erroneous valence-state measurements [88]. Detection limits and quantification accuracy are also constrained: while transition metals can be detected below 100 ppm, the accuracy of valence-state determination is compromised by beam damage, and light elements require higher concentrations for reliable quantification. Electron irradiation can induce phase transformations, meaning that the phase identified by EELS may not represent the native state of the core [88]. Finally, in situ experiments are limited to approximately 2 h due to beam sensitivity. These constraints necessitate rigorous control of electron dose and validation against complementary techniques such as X-ray absorption spectroscopy or Mössbauer spectroscopy (in case of Fe).

#### 2.3.5. Inductively Coupled Plasma Mass Spectrometry (ICP-MS) and Inductively Coupled Plasma Optical Emission Spectrometry (ICP-OES)

ICP-MS and ICP-OES are elemental analysis techniques that enable the determination of chemical element concentrations in a sample. The operating principle is based on introducing the sample in solution form into a plasma source. In the case of mass spectrometry, the number of ions is measured according to their mass-to-charge ratio, whereas in optical emission spectrometry, the intensity of optical emission at characteristic wavelengths is recorded. The sensitivity of ICP-MS is generally two to three orders of magnitude higher than that of ICP-OES; however, the detection limits of ICP-OES are also element-dependent and, for many elements relevant to ferritin loading, fall within the low-ppb to ppm range, which is often sufficient for assessing the degree of loading. It should be noted that detection limits depend strongly on the element, analytical wavelength, sample matrix, and instrument configuration, and therefore cannot be defined as a single universal range. Both methods have become widely adopted for the analysis of metal-loaded ferritin nanoparticles [20,21,90].

In a study by Cosottini et al. [20], ICP-OES was employed for the quantitative determination of gold content in the HuHf@AuTM bioconjugate, i.e., ferritin nanoparticles loaded with aurothiomalate (AuTM). The analysis revealed that, on average, approximately 70 gold atoms were bound per ferritin nanoparticle, corresponding to about 2.9 gold atoms per each of the 24 protein subunits. These data confirmed the successful formation of a stable bioconjugate with a controlled and reproducible drug-loading level and enabled precise characterization of the complex stoichiometry, a critical parameter for assessing its potential therapeutic efficacy. The obtained values were consistent with electrospray ionization mass spectrometry (ESI-MS) results and indicated a high degree of apoferritin conversion into the target conjugate without significant gold release during purification (dialysis), demonstrating the strength of AuTM binding to the protein.

In a further study [90], the Cu_2_O@Fn nanoplatform was quantitatively characterized by ICP-MS, which revealed that approximately 120 copper atoms were incorporated per ferritin particle. Additionally, ICP-MS was used to evaluate intracellular accumulation and biodistribution: in HL-60 cells, the copper concentration following treatment with Cu_2_O@Fn was approximately threefold higher than that observed with free Cu_2_O nanoparticles, whereas in mouse organs, copper levels in the Cu_2_O@Fn group were lower. These findings confirmed targeted delivery mediated by interaction with the CD71 receptor and a narrower off-target distribution profile. Furthermore, the pharmacokinetics of the formulation were characterized by monitoring blood copper dynamics over time.

Thus, ICP-MS and ICP-OES provide highly accurate and reproducible quantitative data essential for correlating analytical parameters with biomedical outcomes, ranging from therapeutic efficacy to pharmacokinetic profiling. However, calculation of the number of metal atoms per ferritin particle also requires an independent and reliable determination of ferritin or protein concentration. Sample digestion, blank correction, calibration strategy, recovery, contamination control, and measurement uncertainty should also be taken into account, as these parameters are important for interlaboratory comparability. The application of ICP-MS and ICP-OES in combination with complementary techniques (e.g., ESI-MS or EDX) enables comprehensive verification of both loading levels and the stability of bioconjugates, which is critically important for protocol standardization and the subsequent clinical translation of ferritin-based nanocarriers.

#### 2.3.6. Mössbauer Spectroscopy

Mössbauer spectroscopy is an analytical technique based on the Mössbauer effect, which involves the resonant absorption of gamma radiation by atomic nuclei without energy loss due to recoil [91]. A key requirement of this method is that the nuclei in the source and in the sample must be identical; therefore, its application is limited to a relatively small number of isotopes, primarily Fe, Co, Sn, and Ir.

In the context of ferritin nanoparticle research, Mössbauer spectroscopy serves as a unique tool for investigating the state of iron within the core. It enables the determination of the oxidation state of iron atoms (Fe^2+^, Fe^3+^, or mixed-valence states), elucidation of the local atomic environment, including coordination geometry and symmetry, differentiation of iron-containing phases (e.g., ferrihydrite, magnetite, and hematite) within the core, assessment of the degree of crystallinity and the presence of amorphous regions, and investigation of the dynamics and mobility of iron atoms within the core structure. Early Mössbauer studies of ferritin demonstrated that the ferritin core contains Fe^3+^ in a high-spin state coordinated by oxygen in an octahedral geometry [92]. These findings were subsequently confirmed and extended by temperature-dependent Mössbauer measurements, which allowed the magnetic transitions within the ferritin core to be characterized [44]. More recent studies have employed Mössbauer spectroscopy to evaluate the stability of the iron-containing core under various treatment conditions, such as thermal denaturation or chemical modification [93]. Additionally, the method has been applied to compare the core structure of native and recombinant ferritins, revealing differences in the iron oxidation state and its local coordination environment [94].

However, Mössbauer spectroscopy has several limitations: it is applicable only to elements with Mössbauer-active isotopes (primarily Fe), requires relatively high concentrations of the target element to obtain high-quality spectra, and spectral interpretation can be complicated for complex, heterogeneous systems. Consequently, this method is of limited utility for ferritin nanoparticles loaded with other metals and is entirely inapplicable to ferritin loaded with organic compounds.

#### 2.3.7. Circular Dichroism Spectroscopy

Circular dichroism (CD) spectroscopy is an analytical technique based on the differential absorption of left- and right-circularly polarized light by a sample [95]. It is particularly sensitive to chiral (asymmetric) structures, including proteins, nucleic acids, and other biomolecules with spatial asymmetry. As polarized light passes through the sample, the difference in absorption between the two polarization states is recorded as a CD spectrum. The shape and intensity of the spectral bands are directly related to the secondary structure of proteins: characteristic minima at approximately 208 nm and 222 nm indicate the presence of α-helices, a minimum near 217 nm is associated with β-sheets, and a broad band around 200 nm corresponds to disordered or random-coil regions [96]. It should be noted that secondary-structure percentages derived from CD spectra depend on the spectral deconvolution approach, reference dataset, wavelength range, protein concentration, and optical path length; these limitations should be taken into account when interpreting relatively small differences in calculated secondary-structure content.

CD spectroscopy was used to evaluate the effects of pH variations and Ag^+^/Cu^2+^ binding on the secondary structure of *Dendrorhynchus zhejiangensis* ferritin (DzFer) [97]. Analysis of the far-UV ellipticity (190–260 nm) revealed that the protein retains a high α-helical content (90.8 ± 0.2%) over the pH range of 4–10, which is typical of the native ferritin structure. At extreme pH values (2 and 12), the α-helical content decreased sharply, while the proportion of β-sheets and disordered regions increased, indicating protein denaturation. Furthermore, the binding of silver ions induced more pronounced changes in the CD spectrum, reducing the α-helical content by 17%, compared to a reduction of only 4.2% observed with Cu^2+^. This indicates a stronger structural impact of Ag^+^ on ferritin, consistent with its greater ability to suppress the ferroxidase activity of DzFer compared to copper ions.

In the context of ferritin-based nanocarriers, CD spectroscopy is particularly valuable for verifying that the encapsulation of therapeutic or diagnostic agents does not compromise the structural integrity of the protein cage [97]. This method also enables comparative analysis of the effects of different metal ions, pH conditions, or chemical modifications on protein conformation, thereby providing essential information for optimizing loading protocols and ensuring the stability of the final formulation. Furthermore, CD spectroscopy can be used to monitor temperature-induced unfolding and to assess the reversibility of ferritin reassembly following pH-mediated cargo loading [98].

### 2.4. Other Methods

#### 2.4.1. Dynamic Light Scattering (DLS) and Zeta Potential

Dynamic light scattering (DLS) is one of the most widely used techniques for the physicochemical characterization of nanoscale systems in solution [99]. The method is based on the registration of temporal fluctuations in scattered light intensity caused by the Brownian motion of particles. The rate of these fluctuations is determined by the translational diffusion coefficient, which is subsequently converted into the hydrodynamic diameter (Dh) using the Stokes–Einstein equation [100]. In contrast to electron microscopy techniques, which provide size information in the dry state, DLS characterizes particles in their native hydrated form.

A key advantage of DLS is the ability to perform rapid, non-destructive analysis under near-physiological conditions. In addition to Dh, the method enables the determination of the Z-average (hydrodynamic size obtained from cumulant analysis of the autocorrelation function), the polydispersity index (PDI), and the zeta potential (ζ-potential) using electrophoretic light scattering. It also allows indirect assessment of aggregation behavior, interparticle interactions, colloidal stability, and the kinetics of structural rearrangements [30]. The Z-average represents an intensity-weighted mean value and is considered the most reproducible parameter for comparative analysis of monodisperse systems. The PDI, derived from cumulant analysis, reflects the breadth of the size distribution and serves as an indicator of sample heterogeneity, although it should be interpreted with caution. The ζ-potential may indicate electrostatic stabilization, since high absolute values of surface charge can reduce the probability of aggregation; however, ζ-potential alone cannot universally predict colloidal stability, particularly in high-ionic-strength or protein-containing media, where steric stabilization, protein corona formation, and charge-screening effects may also play important roles.

For native ferritin, DLS is most commonly used to confirm the integrity of the quaternary structure and to assess system homogeneity [101]. Typically, the hydrodynamic diameter of native ferritin ranges from 12 to 13 nm, while apoferritin exhibits slightly larger values, which is attributed to more pronounced hydration and changes in the density of the protein shell [102,103].

For ferritin nanocarriers loaded with low-molecular-weight compounds, DLS enables the detection of size changes resulting from cargo encapsulation. For example, the incorporation of doxorubicin is usually accompanied by an increase in Dh to 16–20 nm, reflecting not only the presence of molecules within the internal cavity but also the possible interaction of part of the drug with the inner surface of the protein cage [104]. Similar effects are observed upon cisplatin loading, where the size change is often accompanied by a shift in ζ-potential due to the coordinative interaction of platinum with amino acid residues [105]. Metal-loaded systems, such as magnetoferritin, are of particular interest because the mineralization of the internal cavity with iron oxides significantly alters the density and internal organization of the nanoparticle [106,107]. In such cases, DLS typically detects an increase in Dh and a rise in PDI, reflecting higher structural variability. A similar approach is also applied to the analysis of ferritin loaded with gold, platinum, or palladium species, where DLS allows indirect monitoring of the degree of mineralization and nanocomposite stability [78,108,109].

In addition to monitoring cargo loading, DLS plays an important role in studying the dynamics of ferritin self-assembly. Changes in pH, ionic strength, or temperature can induce subunit dissociation and subsequent reassociation, which is accompanied by changes in Dh [101,110]. At pH 2–3, the protein cage undergoes disassembly with the formation of intermediate oligomeric states, whereas a return to neutral pH initiates reassembly [111,112]. DLS allows these processes to be monitored in real time, making the method particularly valuable for optimizing encapsulation protocols.

Despite its widespread use, DLS has several fundamental limitations. One of the most significant is the dependence of scattering intensity on the sixth power of the particle diameter (I ~ d^6^) [113]. This means that even a small number of aggregates can dominate the signal and substantially distort the size distribution. For ferritin, which is prone to reversible aggregation under changes in pH or elevated ionic strength, this represents a serious analytical challenge. An additional limitation is the assumption of spherical particle geometry inherent in the Stokes–Einstein model. Although ferritin normally has a quasi-spherical shape, surface modification, partial denaturation, or asymmetric loading may alter its hydrodynamic behavior, leading to discrepancies between the measured size and the actual morphology. Moreover, DLS cannot distinguish between empty and loaded nanoparticles if the difference in Dh falls within the instrumental error.

A particularly critical limitation for ferritin is its small particle size. Particles of approximately 12 nm produce a relatively weak scattering signal, making the results sensitive to protein concentration, the presence of dust, microbubbles, and even minor variations in solvent viscosity. At low concentrations, the signal-to-noise ratio decreases, whereas at high concentrations, multiple scattering effects arise, leading to overestimation of the measured sizes [114].

Thus, DLS remains one of the most informative and versatile methods for the primary characterization of ferritin nanoparticles, enabling rapid assessment of their size, homogeneity, surface charge, and stability in solution. However, the high sensitivity of the method to aggregates, its limited structural resolution, and its dependence on experimental conditions require careful data interpretation and confirmation by complementary analytical approaches. The combination of DLS with high-resolution techniques provides the most comprehensive understanding of the structural organization and functional state of ferritin nanoparticles.

#### 2.4.2. Atomic Force Microscopy

The possibility of visualizing the iron cores of ferritin by scanning tunneling microscopy (STM) was first demonstrated by Yang et al. in 1992 [115]. The data obtained by this group were consistent with previously known structural parameters; in particular, the lattice constant of the ionic crystals was determined to be 4.9 ± 0.5 Å. Since STM is based on the detection of a tunneling current between a sharp tip and a conductive sample, its application to biological materials remains very limited. Following the introduction of the atomic force microscope (AFM) concept in 1986 [116], direct visualization of non-conductive materials, including biological objects, became possible and was realized for ferritin as early as 1992 [117]. Based on structural analysis, Ohnishi et al. suggested that the observed dipole-like structural feature corresponds to a channel for iron atoms on the surface of the polypeptide shell along the fourfold symmetry axis [117].

In its basic configuration, AFM is a high-resolution surface imaging technique (down to atomic resolution) based on the detection of interaction forces between a nanoscale probe (cantilever) and the sample surface [116]. AFM is suitable for studying both conductive and non-conductive materials, including biological objects, polymers, and other soft materials [118]. With the development of AFM, it has become possible not only to visualize ferritin but also to investigate its protein shell, core, and dynamic behavior under various environmental conditions.

**Figure 5:**
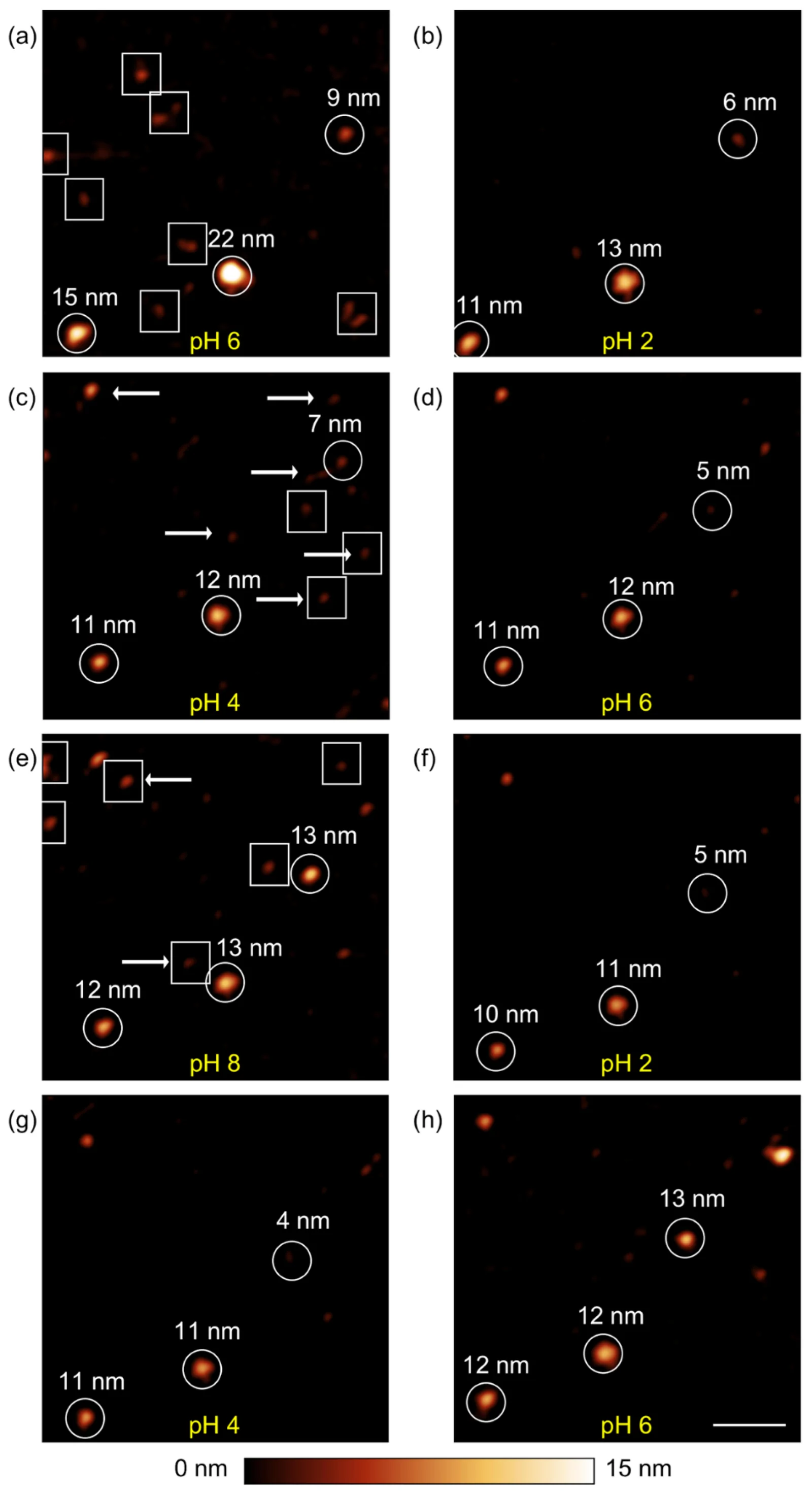
AFM topography images of ferritin nanoparticles adsorbed on a mica substrate measured in deionized water with adjusted pH value in the following order: (**a**) pH 6 (**b**) pH 2 (**c**) pH 4 (**d**) pH 6 (**e**) pH 8 (**f**) pH 2 (**g**) pH 4 (**h**) pH 6. The repeated pH values—(**a**), (**d**), and (**h**) at pH 6; (**b**) and (**f**) at pH 2; (**c**) and (**g**) at pH 4—are intentional and demonstrate the reversibility of ferritin disassembly and reassembly upon cycling of the pH. Particles that vanished in the next step are enclosed by a square. Particles that assembled and did not exist in the previous step are marked by an arrow. Particles present through the whole experiment but vary in size are marked with a circle. Their sizes in nm are designated by numbers. Scale bar is 100 nm. Adapted from ref. [101].

In a study by Johnson et al. [119], the behavior of an adsorbed layer of ferritin nanoparticles in a liquid environment was investigated as a function of key parameters, including ionic strength, pH, and the type of substrate surface. Performing measurements directly in the aqueous phase minimizes artifacts associated with sample drying and provides a more accurate description of processes under native conditions. Stühn et al. [101] employed liquid and magnetic AFM to examine how the structure of the ferritin and apoferritin protein shells changes with pH, specifically monitoring variations in magnetic and mechanical properties. It was established that at reduced pH (from 6 to 2), the particle size decreased, indicating complete disassembly of the protein shell, whereas the size was restored upon increasing the pH (Figure 5).

Thus, AFM can be effectively applied to investigate the dynamic behavior of ferritin nanoparticles, particularly through experiments in liquid media under changing external conditions, such as variations in pH or ionic strength [119,120].

#### 2.4.3. Analytical Ultracentrifugation (AUC)

Analytical ultracentrifugation (AUC) is a solution-phase technique based on first principles that monitors the sedimentation and diffusion of particles under a high-intensity centrifugal field. Unlike dynamic light scattering, where large aggregates disproportionately dominate the scattering signal, AUC registers all sedimenting species in a fractionation mode, providing distributions of sedimentation coefficients, diffusion coefficients, molar masses, particle sizes, and densities [121]. Sedimentation velocity experiments measure the rate of boundary movement under high centrifugal force, which depends on the mass and frictional properties of the analyte, while sedimentation equilibrium experiments performed at lower rotor speeds provide access to molecular weight distributions and interaction parameters [121].

For ferritin nanocarriers, AUC is particularly valuable because the method is sensitive not only to particle size but also to density. This allows characterization of cargo loading heterogeneity independently of the nature of the encapsulated agent. For inorganic cores (oxides of iron, gadolinium, lutetium, manganese, titanium, etc.), AUC registers the distribution of sedimentation coefficients, which directly reflects variations in metal content and mineral core size among molecules in the sample. For organic cargoes (cisplatin, doxorubicin, nucleic acids), the method enables assessment of binding stoichiometry and loading homogeneity, since the incorporation of molecules into the internal cavity alters the density and hydrodynamic properties of the nanocapsule.

A key application of AUC to ferritin was demonstrated by May et al. [122], who used sedimentation velocity experiments to measure mineral core size distributions in human H-chain apoferritin (apoHuHF) and horse spleen apoferritin (apoHoSF). The study revealed that the homopolymer human H-chain ferritin produces more uniform core sizes (diameters ranging from 3.8 ± 0.3 to 6.2 ± 0.3 nm) than the heteropolymer horse spleen ferritin, in which subpopulations of molecules with varying iron content were observed. The study also reported comprehensive physicochemical parameters for apoHuHF (M = 506,266 g/mol, $\overset{-}{\nu }$ = 0.724 mL/g, s_20_,w = 18.3 S, D_20_,w = 3.18 × 10^−7^ cm^2^/s) and apoHoSF (M = 484,120 g/mol, $\overset{-}{\nu }$ = 0.735 mL/g, s_20_,w = 17.0 S, D_20_,w = 3.21 × 10^−7^ cm^2^/s).

A particularly elegant extension of AUC for ferritin analysis was developed by Cölfen and Völkel [123], who addressed the difficulty posed by the superposition of size and density distributions in ferritin. Because ferritin is an organic-inorganic hybrid colloid with varying cargo content and a tendency to oligomerize, precise analysis by AUC alone is challenging. The authors combined the sedimentation coefficient distribution from AUC with the diffusion coefficient distribution obtained from flow-field flow fractionation (Fl-FFF) to yield the buoyant molar mass of oligomers in the mixture. This hybrid approach extended the information content of each individual method and demonstrated that ferritin oligomers up to the pentamer adopt a globular solution structure.

Beyond iron-containing cores, AUC has been successfully applied to ferritin loaded with other agents. Amos et al. [124] used AUC to characterize the mineralization of titanium dioxide (TiO_2_) within H-chain homopolymers of human ferritin. Incremental addition of titanium(III) citrate resulted in the formation of amorphous TiO_2_ particles (1.5–6.5 nm) inside the protein cage. Notably, with up to 1000 equivalents of metal, there was no change in the protein hydrodynamic radius or diffusion constant. However, a systematic shift in the sedimentation coefficient was observed, which confirmed mineralization within the protein core rather than on the surface. This example demonstrates the sensitivity of AUC to internal cargo loading even when external dimensions remain unchanged.

In another study, Falvo et al. [125] employed AUC to characterize an antibody-drug conjugate based on human ferritin encapsulating cisplatin and conjugated to a monoclonal antibody (HFt-Pt-Ep1). Size-exclusion chromatography could not reliably estimate the molecular weight of the conjugate, so the authors used sedimentation velocity experiments. HFt-Pt sedimented as a homogeneous peak at 15.1 S (calculated mass ~500 kDa), while HFt-Pt-Ep1 gave a single peak at ~25.6 S (calculated mass ~900 kDa), corresponding to approximately three antibody molecules per ferritin cage. Thus, AUC enabled determination of the conjugation stoichiometry that could not be established by other methods.

The limitations of AUC for ferritin analysis include the requirement for relatively high sample concentrations, the long duration of experiments, and the complexity of data interpretation for polydisperse systems. Nevertheless, AUC remains one of the few methods capable of quantitatively characterizing the heterogeneity of cargo loading and the oligomeric state of the protein shell in solution, making it an indispensable tool for controlling the reproducibility of ferritin nanocapsule synthesis and assessing the homogeneity of the resulting preparations [121,122].

Summary table (Table 1) of the applicability of various analytical methods for the characterization of ferritin nanoparticles is provided below.

## 3. Perspectives in the Analysis of Loaded Ferritin Nanoparticles

Analysis of the current state of research reveals a fundamental issue hindering the clinical translation of ferritin-based nanocarriers: the vast majority of studies remain fragmented and incomplete. In many cases, research groups focus on a single isolated aspect of the life cycle of loaded ferritin without advancing the study to the stage required for evaluating its therapeutic or diagnostic potential. The most frequently encountered scenarios include successful loading without functional assessment, or a focus limited either to the physicochemical process itself or to biological aspects alone.

An example of the first scenario is represented by studies that conclude at the stage of demonstrating successful cargo encapsulation [9,33]. Thus, Sánchez et al. [33] used a combination of TEM, energy-dispersive X-ray spectroscopy (EDX), and ICP-MS to demonstrate that gadolinium oxide (Gd_2_O_3_) nanoparticles were quantitatively loaded into the apoferritin cavity (~1700 Gd atoms per molecule), while the integrity of the protein shell was confirmed by native polyacrylamide gel electrophoresis (PAGE) and negative-stain TEM. Moreover, the authors measured the relaxivity of the resulting nanoparticles, showing that longitudinal relaxivity (r_1_) exceeded that of commercial Gd chelates by 10–25-fold and transverse relaxivity (r_2_) by approximately 70-fold, and confirmed complex stability (only 5% Gd loss over 7 days). Nevertheless, the study did not progress further: no cytotoxicity assessment, cellular uptake studies, *in cellulo* MRI visualization, or, most critically, in vivo experiments were performed.

In the second scenario, the focus is shifted either exclusively to the physicochemical aspects of loading/release in model buffer systems or, conversely, to the final biological effect without adequate understanding of the nanoplatform structure [35,126]. Some studies remain at an intermediate stage, for example, concluding after in vitro investigations [127].

Examples of completed studies include [15,31,32,36,128]. In the work by Wang et al. [15], the following conditions were fulfilled: (1) determination of loading efficiency and complex stability—the biomimetic synthesis of CuS within the ferritin cavity was confirmed by TEM and EDX mapping, while the stability of radiolabeled ^64^CuS–Fn NCs in serum was verified by radio-TLC (no free copper release over 24 h); (2) assessment of protein shell integrity after loading—DLS confirmed the preservation of nanoparticle monodispersity, and UV–Vis–NIR spectroscopy revealed the appearance of intense absorption characteristic of the formed CuS core; (3) investigation of biodistribution and tumor accumulation—performed using three-dimensional photoacoustic imaging (PAI) and positron emission tomography (PET) with ^64^Cu, enabling quantitative assessment of tumor uptake dynamics (up to ~10 percentage of injected dose per gram (% ID/g) at 8 h) and excretion; and, most importantly, (4) evaluation of therapeutic efficacy in a preclinical model—PAI/PET-guided photothermal therapy achieved 100% elimination of U87MG tumors without recurrence, and histological examination (H&E) of major organs at 15 and 30 days confirmed the absence of systemic toxicity. Such completeness of data provides a particularly strong basis for progressing to clinical trials, although the scope of required characterization, biodistribution, efficacy, and toxicity data may vary depending on the intended product, indication, development stage, and regulatory strategy.

To overcome the described gap, the development of comprehensive analytical protocols tailored to the nature of the encapsulated cargo is required.

For inorganic cores, the primary objective is to establish a clear relationship between the synthesis method, the physicochemical properties of the core, and the functional response. A comprehensive approach should include: identification of phase composition and crystallinity (X-ray diffraction (XRD) allows unambiguous determination of the crystalline phase, e.g., distinguishing ferrihydrite (5Fe_2_O_3_·9H_2_O) from magnetite (Fe_3_O_4_) or maghemite (γ-Fe_2_O_3_); for X-ray amorphous samples (e.g., phosphate cores) or nanoparticles smaller than 2 nm, HRTEM combined with electron nanodiffraction is the method of choice); analysis of chemical and electronic state (X-ray photoelectron spectroscopy (XPS) and X-ray absorption spectroscopy (X-ray absorption near-edge structure (XANES)/EXAFS) are indispensable for determining the oxidation state of metal atoms and their local environment; for example, XPS can distinguish Pt(0) from Pt(II) in the core, while EXAFS can confirm the core structure as “planes of Fe separated by O/OH layers,” which is critical for understanding release mechanisms); and quantitative elemental analysis (ICP-MS or ICP-OES serve as the “gold standard” for determining the average number of metal atoms per ferritin particle, a key parameter for dosage calculation and reproducibility). For visualization of the spatial distribution of elements within the cavity and exclusion of surface adsorption, TEM-EDX mapping is employed.

For organic agents (doxorubicin, cisplatin, nucleic acids), analytical priorities shift toward confirming the integrity of the encapsulated molecule and its interaction with the protein shell. This requires: confirmation of encapsulation and binding stoichiometry (ultrafiltration combined with UV–Vis spectrophotometry or HPLC allows assessment of loading efficiency; native mass spectrometry (ESI-MS) can provide the exact mass of the “protein–ligand” complex, determining the number of drug molecules per subunit, as demonstrated for aurothiomalate conjugates [20]); assessment of structural integrity and conformational changes (far-UV circular dichroism (CD) spectroscopy and Fourier-transform infrared (FTIR) spectroscopy enable detection of changes in protein secondary structure after loading; intrinsic tryptophan fluorescence spectroscopy is sensitive to alterations in tertiary structure near the binding site); and analysis of intermolecular interactions (isothermal titration calorimetry (ITC) and surface plasmon resonance (SPR) allow quantitative characterization of the affinity, stoichiometry, and thermodynamics of drug–protein interactions, which is important for predicting stability and release kinetics).

Regardless of the nature of the cargo, the assessment of colloidal stability and protein shell integrity of the final construct under physiologically relevant conditions (e.g., in phosphate-buffered saline (PBS) or medium containing 10% fetal bovine serum (FBS) at 37 °C) is mandatory. For this purpose, DLS (monitoring hydrodynamic diameter and PDI) and TEM (visualization of possible aggregation or degradation) are used over time.

To ensure that successful laboratory results do not remain merely scientific publications but become the basis for clinical trials, research is best structured in accordance with a logic that anticipates regulatory requirements and, where feasible, encompasses the full cycle of «synthesis → characterization → in vitro functional assessment → multimodal in vivo imaging → pharmacokinetics → toxicology.»

## 4. A Stepwise Strategy for Translation

Stage 1: Optimization and standardization (analytical). The primary goal of this stage is the development of a reproducible and scalable synthesis and loading protocol supported by strict acceptance criteria. Based on the multimodal analytical strategy, specifications for critical quality attributes are established: mean hydrodynamic size and PDI, ζ-potential, loading degree (number of atoms/molecules per nanoparticle), preparation purity (absence of free agent as determined by HPLC/ICP-MS in the ultrafiltrate), sterility, and endotoxin content (limulus amebocyte lysate (LAL) test). For each parameter, an acceptable range of values is defined based on several independent sample batches. The outcome of this stage is a “nanopreparation passport” and a draft specification.

Stage 2: Proof of concept in complex biological systems (functional in vitro). The key objective is to demonstrate that the stability and functionality of the nanopreparation are maintained under near-in vivo conditions. This requires investigation of cargo release kinetics under physiologically relevant conditions rather than in simple buffer, for example, in serum, plasma, or cell culture medium at 37 °C, with the choice of biological matrix guided by the intended route of administration, mechanism of action, and expected in vivo environment; assessment of nanoconstruct stability in the selected medium over 24–72 h by DLS and gel filtration; evaluation of cellular uptake (by ICP-MS, confocal microscopy, or flow cytometry) and internalization mechanism (inhibitor analysis) in relevant target cell lines; and direct correlation of the internalized dose with the therapeutic effect (cytotoxicity, apoptosis).

Stage 3: Preclinical validation (functional in vivo). The aim of this stage is to obtain data on efficacy, biodistribution, pharmacokinetics, and toxicity in animal models. At this stage, the previously developed analytical methods become tools for bioanalysis. ICP-MS is used for ex vivo determination of metal content in organs and tissues to construct biodistribution profiles and calculate pharmacokinetic parameters. For radioactive labels, an appropriate radionuclide-specific nuclear imaging modality—such as single-photon emission computed tomography/computed tomography for gamma-emitting radionuclides or positron emission tomography for positron-emitting radionuclides—enables non-invasive dynamic monitoring of tumor and organ accumulation and dosimetric calculations. Immunohistochemistry (IHC) with anti-ferritin antibodies can confirm nanoparticle localization in tissues. In parallel, acute and chronic toxicity are assessed in accordance with good laboratory practice (GLP) principles.

Stage 4: Preparation of the regulatory dossier. At this stage, the accumulated scientific data are transformed into a format required by regulatory agencies (e.g., FDA, EMA). This requires systematization of all critical parameter data, validation of analytical methods, and preparation of the pharmaceutical section of the dossier (CMC—Chemistry, Manufacturing and Controls). At this point, the absence of data fragmentation becomes critical: characterization protocols must be presented not merely as descriptions but as standard operating procedures with demonstrated accuracy, specificity, and reproducibility.

Only such a continuous cycle, in which each analytical parameter is directly linked to biological function and, ultimately, to clinical outcome, will enable ferritin-based nanocarriers to overcome the “valley of death” of translational research and become real biomedical products.

## 5. Conclusions

The analysis presented in this review leads to a central conclusion: the principal obstacle to the clinical translation of ferritin-based nanocarriers is not the absence of analytical techniques, but the absence of a unified framework that links analytical parameters to biological outcomes and regulatory requirements. The analytical toolbox is already broad and continues to expand, yet its application remains fragmented, and the resulting data are rarely comparable across laboratories or directly translatable into quality specifications.

Three key recommendations follow from this. First, characterization should be multimodal by default: no single method can capture the structural, chemical, and functional complexity of a loaded ferritin cage, and the combination of complementary techniques—for example, TEM with XRD, or ICP-MS with Raman spectroscopy—should be treated as the minimum standard rather than an optional refinement. Second, acceptance criteria should be organized in a tiered manner: universal platform-level criteria (preservation of the 24-meric assembly, absence of aggregation, sterility and endotoxin limits, absence of free cargo, stability in serum, and reproducibility across independent batches) combined with cargo-specific and application-specific criteria, since the diversity of ferritin systems—from MRI contrast agents to radiotherapeutics, photosensitizers, and nucleic acid carriers—makes a single uniform specification neither feasible nor scientifically justified. Third, each analytical parameter should be explicitly correlated with a biological readout so that physicochemical characterization becomes predictive of efficacy, biodistribution, and safety rather than an end in itself.

Implementing these recommendations would transform ferritin characterization from a collection of independent measurements into a coherent, reproducible, and regulatorily compatible workflow—a necessary condition for ferritin-based theranostic systems to move beyond proof-of-concept studies and become viable biomedical products.

## Figures and Tables

**Table 1:** Applicability of analytical methods for the characterization of loaded ferritin nanoparticles.

Method	Application	Applicability to Compounds	Quantitative Capability	Detection Limit	Key Limitations	References
Transmission electron microscopy (TEM)	Visualization of the shape and size of ferritin particles and their cores; assessment of impurities and agglomeration.	Inorganic (metals, oxides: Fe_3_O_4_, Ga_2_O_3_, Lu_2_O_3_, MnO_2_). Organic (drugs, nucleic acids, peptides)—limited: weak contrast for light elements (C, N, O); organic cores are not directly visualized.	Semi-quantitative (size, morphology, loading degree by counting)	Single-particle detection; depends on Z-contrast	Sample drying artifacts; organic cores not visualized; limited statistics; beam damage for organic specimens	[16,41,42,43,44,48,49,50,51,52]
X-ray diffraction (XRD)	Qualitative and quantitative phase analysis; determination of crystal structure, lattice parameters, and core crystallinity.	Inorganic—primary method for identifying phases (magnetite, ferrihydrite, etc.). Organic—not applicable: amorphous organic molecules do not produce distinct diffraction patterns.	Quantitative for crystalline phase content	~1–5% crystalline phase detection; requires ≥2 nm crystallites	Amorphous or very small (<2 nm) cores not detectable; protein shell must be removed by thermal treatment; no spatial information	[55,56,64]
Energy-dispersive X-ray spectroscopy (EDX)	Elemental confirmation in the core; assessment of local elemental distribution and ratios	Inorganic—applicable. Organic—limited: lower sensitivity to light elements.	Semi-quantitative (elemental ratios); quantitative with standards	~0.1–1 wt% for transition metals; worse for light elements (C, N, O)	Poor sensitivity for light elements; overlapping peaks; X-ray generation volume exceeds particle size in SEM-EDX; no chemical-state information	[15,81,82,83]
Electron energy-loss spectroscopy (EELS)	Elemental and chemical-state analysis at near-atomic resolution; determination of oxidation state and local coordination environment; spatially resolved chemical mapping in STEM-HAADF mode.	Inorganic (transition metals, noble metals, metal oxides)—applicable. Light elements (C, N, O, S, Se)—applicable. Organic—limited due to beam damage.	Quantitative (elemental ratios, oxidation state via ELNES); atom counting possible	<100 ppm for transition metals; ~0.2% for light elements in matrix	Severe beam damage (reduction of Fe^3+^ to Fe^2+^, ligand loss); phase transformations; requires ultrathin specimens	[44,51,84,85,86,87,88,89]
X-ray photoelectron spectroscopy (XPS)	Surface and near-surface elemental analysis; determination of chemical states (oxidation state, bonding environment).	Inorganic (metals, oxides)—applicable: binding energy shifts reflect chemical state. Organic—not recommended: prolonged X-ray exposure often degrades organic molecules.	Quantitative for surface composition (at%); chemical-state quantification	~0.1–1% at for most elements; surface-sensitive (5–10 nm depth)	Surface-only information; organic degradation under X-rays; not representative of bulk core; requires vacuum	[77,78,79]
Mössbauer spectroscopy	Detailed characterization of iron in the core: oxidation state (Fe^2+^/Fe^3+^), local coordination and symmetry, iron-containing phase identification, degree of crystallinity, amorphous regions, atomic dynamics.	Only for iron (and other Mössbauer-active isotopes: Co, Sn, Ir). Not applicable to other metals or organic molecules.	Quantitative (Fe^2+^/Fe^3+^ ratios, phase fractions, magnetic parameters)	Requires ~0.1–1 mg of iron; ~1–5% phase detection	Only Mössbauer-active isotopes; high sample concentration required; complex spectral interpretation for heterogeneous systems	[92,93,94]
ICP-MS/ICP-OES	Accurate quantitative elemental analysis (Ga, Lu, Fe, Au, Cu, Pt, etc.) in loaded ferritin and biological samples.	Inorganic—gold standard for quantitative metal loading assessment. Organic—applicable for elemental composition of ligands (e.g., P in nucleic acids), but does not directly confirm encapsulation.	Quantitative (elemental concentration, atoms per particle)	ICP-MS: ppt–ppb range; ICP-OES: ppm–ppb range	Destructive; no spatial or chemical-state information; requires sample digestion; cannot distinguish encapsulated vs. surface-bound cargo	[20,21,90]
Raman spectroscopy	Non-destructive analysis of chemical composition and structure; simultaneous investigation of the mineral core and protein shell (amide bands, aromatic amino acid residues).	Inorganic (oxides, minerals)—applicable: characteristic bands for ferrihydrite, magnetite, maghemite. Organic—applicable: molecular identification and analysis of cargo–shell interactions.	Semi-quantitative (phase identification); quantitative with calibration	~1–10% for mineral phases; depends on laser power and acquisition time	Laser-induced sample damage; fluorescence interference; weak signal for dilute samples; limited spatial resolution (~1 µm)	[73]
FTIR spectroscopy	Analysis of functional groups; confirmation of organic molecule encapsulation; study of interactions between cargo and protein shell.	Organic—primary method. Inorganic—limited: mainly for surface groups of the protein shell or impurities.	Semi-quantitative (functional group identification); quantitative with calibration	~1–5% for major functional groups	Overlapping bands; water interference; limited information on metal cores; requires drying or specific sample preparation	[74]
Circular dichroism (CD) spectroscopy	Assessment of the secondary structure of the ferritin shell (α-helix, β-sheet, disordered content); detection of conformational changes induced by cargo loading or pH variations.	Protein shell—primary method. Core (inorganic or organic)—not applicable: sensitive only to chiral protein structures.	Quantitative (α-helix, β-sheet, disordered content in %)	~5–10% change in secondary structure content	Only reports on protein shell; no core information; requires optically clear solutions; sensitive to buffer absorption	[96,97,98]
Dynamic light scattering (DLS)	Measurement of hydrodynamic diameter, size distribution (PDI), ζ-potential; assessment of colloidal stability and aggregation in solution.	Universal for nanoparticles in solution; useful for monitoring the stability of ferritin–cargo systems under physiological conditions.	Quantitative (Dh, PDI, ζ-potential); intensity-weighted	~1 nm size resolution for monodisperse samples; ~0.1 mg/mL protein concentration	Aggregates dominate signal (I ~ d^6^); no chemical information; cannot distinguish empty vs. loaded if size difference is small; sensitive to dust and bubbles	[78,99,100,101,103,104,105,106,107,108,109,110,111,112,113,114]
Atomic force microscopy (AFM)	Visualization of surface topography; investigation of dynamic behavior (including in liquid media under changing pH, ionic strength); in situ studies of disassembly/reassembly.	Suitable for protein shell; applicable in liquid environments for native-state studies.	Semi-quantitative (size, morphology, mechanical properties); quantitative with calibration	Sub-nanometer vertical resolution; ~1–10 nm lateral resolution	Surface-bound measurements; tip convolution effects; limited to adsorbed particles; no chemical information	[115,116,117,118,119,120]
Analytical ultracentrifugation (AUC)	Determination of sedimentation and diffusion coefficients, molar masses, particle sizes, and densities; characterization of cargo loading heterogeneity and oligomeric state in solution.	Inorganic cores (Fe, Ti, Gd, Lu, Mn oxides)—applicable: sensitive to density changes. Organic cargoes (cisplatin, doxorubicin, nucleic acids)—applicable: enables assessment of binding stoichiometry and loading homogeneity.	Quantitative (*s*, D, M, density distributions); first-principles method	~0.1–1 mg/mL protein; ~1% difference in sedimentation coefficient	Requires relatively high concentrations; long experiment duration; complex data analysis for polydisperse systems; no chemical-state information	[121,122,123,124,125]

## Data Availability

The original contributions presented in this study are included in the article. Further inquiries can be directed to the corresponding author.

## Abbreviations

The following abbreviations are used in this manuscript:

AFM	Atomic force microscopy
CD	Circular dichroism
CMC	Chemistry, manufacturing and controls
cryo-EM	Cryogenic electron microscopy
DLS	Dynamic light scattering
EDS	Energy-dispersive X-ray spectroscopy
EDX	Energy-dispersive X-ray spectroscopy
EPR	Electron paramagnetic resonance
ESI-MS	Electrospray ionization mass spectrometry
EXAFS	Extended X-ray absorption fine structure
FBS	Fetal bovine serum
FTIR	Fourier-transform infrared spectroscopy
GLP	Good laboratory practice
HAADF	High-angle annular dark-field
HPLC	High-performance liquid chromatography
HRTEM	High-resolution transmission electron microscopy
ICP-MS	Inductively coupled plasma mass spectrometry
ICP-OES	Inductively coupled plasma optical emission spectrometry
IHC	Immunohistochemistry
ITC	Isothermal titration calorimetry
LAL	Limulus amebocyte lysate
MRI	Magnetic resonance imaging
PAGE	Polyacrylamide gel electrophoresis
PAI	Photoacoustic imaging
PDI	Polydispersity index
PET	Positron emission tomography
PXRD	Powder X-ray diffraction
SAED	Selected-area electron diffraction
SANS	Small-angle neutron scattering
SAXS	Small-angle X-ray scattering
SC-XRD	Single-crystal X-ray diffraction
SDS	Sodium dodecyl sulfate
SEM	Scanning electron microscopy
SEM-EDX	Scanning electron microscopy with energy-dispersive X-ray spectroscopy
SPECT/CT	Single-photon emission computed tomography/computed tomography
SPR	Surface plasmon resonance
SQUID	Superconducting quantum interference device
STEM	Scanning transmission electron microscopy
STEM-HAADF	Scanning transmission electron microscopy—high-angle annular dark-field
STM	Scanning tunneling microscopy
TEM	Transmission electron microscopy
TEM-EDX	Transmission electron microscopy with energy-dispersive X-ray spectroscopy
TfR1	Transferrin receptor 1
TR-SAXS	Time-resolved small-angle X-ray scattering
UV–Vis	Ultraviolet–visible spectroscopy
XANES	X-ray absorption near-edge structure
XPS	X-ray photoelectron spectroscopy
XRD	X-ray diffraction
